# *Alternaria alternata* Toxins Synergistically Activate the Aryl Hydrocarbon Receptor Pathway In Vitro

**DOI:** 10.3390/biom10071018

**Published:** 2020-07-09

**Authors:** Julia Hohenbichler, Georg Aichinger, Michael Rychlik, Giorgia Del Favero, Doris Marko

**Affiliations:** 1 Department of Food Chemistry and Toxicology, University of Vienna, 1090 Vienna, Italy; julia.hohenbichler@univie.ac.at (J.H.); georg.aichinger@univie.ac.at (G.A.); giorgia.del.favero@univie.ac.at (G.D.F.); 2 Chair of Analytical Chemistry, Technical University of Munich, 80333 Munich, Germany; michael.rychlik@tum.de

**Keywords:** emerging mycotoxins, combinatory effects, synergism, phase I metabolism

## Abstract

*Alternaria* molds simultaneously produce a large variety of mycotoxins, of which several were previously reported to induce enzymes of phase I metabolism through aryl hydrocarbon receptor activation. Thus, we investigated the potential of naturally occurring *Alternaria* toxin mixtures to induce Cytochrome P450 (CYP) 1A1/1A2/1B1 activity. Two variants of an extract from cultured *Alternaria alternata,* as well as the toxins alternariol (AOH), alternariol monomethyl ether (AME), altertoxin I (ATX-I), and altertoxin II (ATX-II), were tested singularly and in binary mixtures applying the 7-ethoxy-resorufin-*O*-deethylase (EROD) assay in MCF-7 breast cancer cells. Sub-cytotoxic concentrations of the two toxin mixtures, as well as ATX-I, ATX-II and AOH, exhibited dose-dependent enhancements of CYP 1 activity. ATX-I and ATX-II interacted synergistically in this respect, demonstrating the two perylene quinones as major contributors to the extract’s potential. Binary mixtures between AOH and the two altertoxins respectively exhibited concentration-dependent antagonistic as well as synergistic combinatory effects. Notably, AME showed no efficacy towards EROD enzyme activity or impact on other toxins’ efficacy. Hence, this study provides insights into synergistic and other combinatory effects of *Alternaria* toxins in natural co-occurrence scenarios in the context of AhR signalling pathway activation in breast cancer cells.

## 1. Introduction

**Figure 1:**
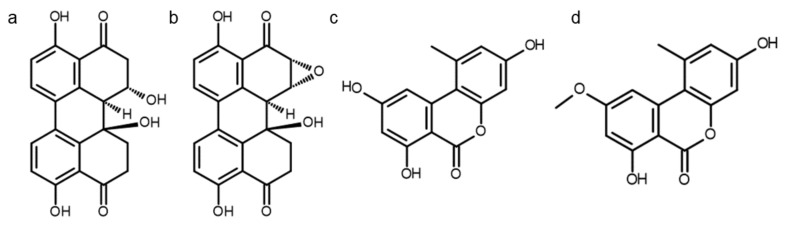
Chemical structures of (**a**) ATX-I, (**b**) ATX-II, (**c**) AOH and (**d**) AME.

The fungal species *Alternaria alternata* is known to infest crops including cereals, fruits, and vegetables [1,2]. A variety of *Alternaria* species have been reported to produce more than 70 secondary metabolites that might co-occur in food and feed. The chemical composition of the resulting highly diverse mixtures depends on several growth conditions [3]. Out of the most prevalent metabolites, several have been characterized chemically and were demonstrated to act as mycotoxins. Thereof, the two dibenzo-α-pyrones alternariol (AOH) and alternariol monomethyl ether (AME) are among the most important *Alternaria* toxins with respect to occurrence [2,4]. Contamination studies on the emerging *Alternaria* mycotoxins such as the perylene quinones altertoxin I (ATX-I) and altertoxin II (ATX-II) are still scarce (Figure 1a,b); yet, in light of their mutagenic potential, they might pose an underestimated health risk [4,5,6]. Frequent crop infestation by *Alternaria* spp., concomitant with simultaneous production of various secondary metabolites, as well as chemical stability during food processing and storage, results in persistent co-occurrence of *Alternaria* toxins [2,4,7,8,9]. A call by the European Food Safety Authority to collect further data on *Alternaria* toxins was followed by a rise in studies investigating nutritional exposure [1,2]. These uncovered co-contaminations of various *Alternaria* toxins in sunflower seed oil, tomato products, infant foods, and diverse fruit and vegetable juices [10,11,12]. Recently, a low abundance of AOH and AME was reported even in breast milk samples [13].

The two dibenzo-α-pyrones AOH and AME (Figure 1c,d) were previously reported to affect cell viability [14] and to moderately induce DNA strand breaks [15]; the latter occurring upon poisoning of topoisomerase II, as demonstrated for AOH [16]. The role of the oxidative stress inducing potential of both dibenzo-α-pyrones towards their genotoxic potential still remains unclear [17,18]. Besides, AOH was reported to exhibit cytotoxic [8], clastogenic [19], and immunomodulatory properties [20,21,22,23] *in vitro* as well as fetotoxic [24] capacities *in vivo* and to possess estrogenic as well as other endocrine disruptive potentials [19,25]. The perylene quinone ATX-II was previously shown to exert genotoxic capacities [26], alter cell membrane biophysical properties of intestinal cancer cells [27], and inhibit the NF-κB pathway in THP-1 derived macrophages [28]. Both ATX-I and ATX-II were reported to possess mutagenic activity in *S. typhimurium*, with a substantially lower potency for ATX-I [5], while the nitrosylation of ATX-I in turn was found to increase mutagenicity against two strains of *Salmonella* [29]. Considering a previous report of ATX-II to be de-epoxidized yielding ATX-I *in vivo* [30], both substances should be implemented in toxicological investigations for a proper risk assessment.

The aryl hydrocarbon receptor (AhR), a basic helix-loop-helix Per-Arnt-Sim transcription factor, can be activated via ligand binding [31]. Upon activation and translocation into the nucleus, the ligand bound to AhR heterodimerizes with the aryl hydrocarbon receptor nuclear translocator (ARNT). This complex regulates the transcription of the cytochromes P450 (CYP) 1A1, 1A2, and 1B1, enzymes involved in xenobiotic phase 1 metabolism, via interaction with the xenobiotic responsive element [32,33]. Additionally, AhR activation has been shown to affect cancer cell proliferation, cell migratory capabilities, and therefore also crucial steps in metastasis and innate as well as adaptive immunity [34,35]. Besides, several CYP isoforms are frequently induced by their own substrates. Hence, prominent ligands of the AhR behave also as inducers of CYP 1A1 activity, such as 2,3,7,8–tetrachlorodibenzo-*p*-dioxin (TCDD) and benzo[a]pyrene (B[a]P), which are suggested to be involved in increased breast cancer risk [36,37,38,39,40]. Furthermore, distinctively elevated CYP 1A1 and CYP 1B1 protein expression as well as EROD activity were previously reported for breast cancer tissues in comparison to adjacent non-tumor tissues [41]. Extensive research has uncovered a complex crosstalk between AhR signalling and estrogen receptor (ER) related pathways in estrogen-sensitive tissues. Diverse mechanisms have been proposed to take part and lead to disparate effects of activation of AhR as well as ER [42,43,44,45]. A standard method used to determine CYP 1A1 enzyme activity induction upon exposure to AhR ligands is the 7-ethoxyresorufin-*O*-deethylase (EROD) assay [46]. The dealkylation of 7-ethoxyresorufin (7-ER) yielding the fluorescent substance resorufin was shown to be primarily exerted through CYP 1A1, followed by CYP 1A2 and CYP 1B1 [47].

AOH and AME were reported to serve as substrates for different CYP enzymes, particularly for CYP 1A1, resulting in the generation of hydroxylated metabolites [48,49]. Previous studiesuncovered the induction of CYP 1A1 expression to be AhR dependent for both dibenzo-α-pyrones in murine hepatoma cells and AOH in human esophageal cancer cells [50,51], whereas the latter turned out to be irrelevant for the genotoxic capabilities of AOH. Although data on the metabolism of altertoxins is scarce, ATX-II was reported to induce CYP 1A1 activity and raise CYP 1A1 transcript levels in KYSE 510 cells [51].

The co-occurrence of toxins may lead to interactions apart from additive combinatory effects, such as positive effect amplifications (synergism) or a reversing impact (antagonism) [52]. A previous combinatory study of AOH and AME revealed a synergistic effect towards cytotoxicity in Caco-2 colon carcinoma cells [53]. Recently, complex *Alternaria* extracts were found to exert anti-estrogenic capacities towards Ishikawa endometrial cancer cells that could partially be ascribed to an ER-AhR/ARNT complex crosstalk [54].

Hence, this study aimed to uncover the potential of naturally occurring mixtures of *Alternaria* toxins to enhance CYP 1 activity and their combinatory effects in this respect applying the EROD assay. For this purpose, two variants of an *Alternaria* extract, the first onward named “complete extract” (CE) gained from inoculation of rice with the *Alternaria alternata* strain DSM62010 [55] and further stripped from ATX-II and STX-III to obtain the second variation named “reduced extract” (RE) [54] were applied. Further, the CYP 1 activating potential of single *Alternaria* toxins, as well as binary mixtures according to the complex extracts, was investigated utilizing the estrogen-sensitive mammary adenocarcinoma cell line MCF-7. Apart from their pronounced genotoxic properties [26], *in vitro* data on ATX-I and ATX-II and their diverse bioactive mechanisms and interactions with co-occurring mycotoxins for proper risk assessment is still lacking. Thus, the present study was performed with special emphasis on the perylene quinone derivatives and their effects in combinations.

## 2. Materials and Methods

### 2.1. Chemicals and Reagents

Cell culture media and supplements were purchased from Gibco Thermo Fisher Scientific (Waltham, MA, USA) and Sigma-Aldrich Chemie GmbH (Steinheim, Germany). Labware for experiments was obtained from Sarstedt AG & Co (Nuembrecht, Germany). AOH, AME, B[a]P, dicoumarol, resorufin, and 7-ER were purchased from Sigma-Aldrich Chemie GmbH (Steinheim, Germany), Triton^®^X 100 and DMSO were purchased from Carl Roth GmbH&Co (Karlsruhe, Germany). ATX-II was gained via extraction from rice infested with *Alternaria alternata* as previously described [26]. ATX-I was synthesized utilizing a loop of conidia of *Alternaria alternata* obtained from a potato leaf as recently reported [56]. A complex extract of *Alternaria alternata* toxins was generated per inoculation of rice with *Alternaria alternata* spores of the strain DSM62010, as recently described [57], and stripped from ATX-II and STX-III according to a previously published procedure [54]. This last step allowed us to generate a second extract which is referred to as “reduced extract”. Both extracts’ compositions were published by Aichinger et al. [54]. Corresponding concentrations of single compounds within the extracts can be found in Table 1.

### 2.2. Cell Line and Culture Conditions

Human mammary breast cancer cells MCF-7 (ATCC, Manassas, VA, USA) were subcultured in DMEM supplemented with 10% fetal calf serum (both Gibco, Thermo Fisher Scientific, Waltham, MA, USA) 100 U/mL penicillin and streptomycin and 0.01 mg/mL insulin solution (Sigma Aldrich) and kept in a humidifier at 37 °C and 5% CO_2_. Cells were passaged every 3–4 days at a confluency of 80% and regularly checked for the absence of mycoplasm contamination. For experiments, cells were seeded in culture medium for 48 h and incubation solutions were prepared using an experimental medium. The experimental medium was comprised of DMEM without phenol red (Gibco, Thermo Fisher Scientific, Waltham, MA, USA), 100 U/mL penicillin and streptomycin and 10% dextran treated charcoal stripped fetal calf serum (Thermo Fisher Scientific, Waltham, MA, USA). Stock solutions of incubation conditions were prepared in DMSO and corresponding solvent controls were prepared accordingly applying 1% of DMSO to the experimental medium.

#### 2.3. 7-Ethoxyresorufin-O-deethylase Enzyme Activity Assay

For the EROD assay, 60,000 cells/well were seeded into 24-well plates and grown for 48 h. For incubation, cells were kept in incubation solutions for 24 h. The EROD assay was carried out as previously described [58]. Briefly, medium containing 5 µM 7-ER and 10 µM Dicoumarol was applied to the cells for 30 min. Afterwards, the enzymatic reaction was stopped by removing the supernatant and the fluorescence readout was obtained from a BioTek Cytation^TM^ 5 plate reader at 535 nm_ex_/595 nm_em_. The concentration of resorufin was calculated with the help of a simultaneously measured standard curve. Subsequently, the protein contents were assessed applying a BCA assay (Thermo Fisher Scientific) according to the manufacturers’ protocol. For calculations of enzyme activity, the respective resorufin concentrations were normalized to protein contents. Also, 1 µM B[a]P served as the positive control for enzyme activity induction, and 1 nM 17-β-estradiol (E2) served as the control for any estrogenic impact on EROD activity.

#### 2.4. Cell Metabolic Activity Assay

In order to measure cell metabolic activity, 8000 cells/well were seeded into 96-well plates and grown for 48 h. This was followed by a 24 h incubation period in experimental media containing incubation stock solutions. Solvent control contained the respective amount of DMSO (1 %) and 0.1 % Triton X served as the negative control. Afterwards, a 1:10 dilution of CellTiter-Blue^®^ 10x concentrated reagent was prepared in DMEM and cells were incubated applying this solution for 1 h. Subsequently, the fluorescence of supernatants was measured using a BioTek Cytation^TM^ 5 plate reader at 560 nm_ex_/590 nm_em_.

#### 2.5. Statistical Analysis

For calculations of combinatory effects that differ from an additive effect of the single compounds, the combination index theorem as described by Chou and Talalay [52,59] was applied to determine interactions concerning the EROD enzyme activity. Statistical and graphical analysis was conducted using OriginPro^®^ 2020. To ensure the normal distribution of the data, a Shapiro-Wilk normality test was applied. Outliers were determined via a Nalimov outlier test and excluded from the data prior to further calculations. Significant differences were calculated utilizing one-way ANOVA coupled to a Bonferroni post-hoc test whenever possible or Student’s *t*-test applying * *p* < 0.05, ** *p* < 0.01, and *** *p* < 0.001.

## 3. Results

### 3.1. EROD Enzyme Activity Induction by Complex Mixtures and Single Toxins

**Figure 2:**
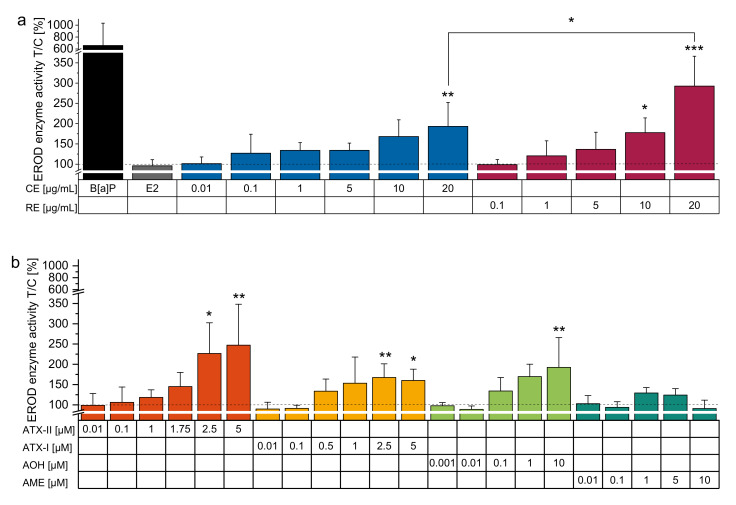
EROD assays with *Alternaria* extracts and single toxins. Enzyme activity measurements (pmol resorufin/mg protein*min) presented as means + SD normalized to solvent control (1% DMSO) of at least 3 individual experiments. Enhancement of EROD enzyme activity after 24 h of incubation applying (**a**) B[a]P (1 µM), E2 (1 nM), CE (0.01 µg/mL–20 µg/mL), RE (0.1 µg/mL–20 µg/mL) and (**b**) single compounds ATX-II (0.01 µM–5 µM), ATX-I (0.01 µM–5 µM), AOH (0.001 µM–10 µM) and AME (0.01 µM–10 µM). Significant differences to the NOEL were calculated by one-way ANOVA, followed by Bonferroni post-hoc testing, and are highlighted by “*” (*p* < 0.05), “**” (*p* < 0.01) and “***” (*p* < 0.001).

EROD enzyme activity assay was conducted applying the complex mixtures of *Alternaria* toxins to test their potential to activate the AhR signalling pathway. Both variants of the extract led to a dose-dependent increase in enzymatic activity. Significant enhancements in EROD activity were reached at concentrations of 20 µg/mL for both the CE and the RE, with a significantly higher induction observed for the RE (Figure 2a).

In order to investigate possible compounds contributing to the overall EROD enzyme activity induction of the complex extract, we tested the single compounds ATX-I, ATX-II, AOH, and AME. Enhancements of EROD activity showed dose-dependency with the most potent effects starting at concentrations of 2.5 µM for ATX-I and ATX-II and at 10 µM for AOH. Strikingly, none of the concentrations applied for AME led to strong enzyme activity inductions (Figure 2b).

### 3.2. Combinatory Effects on EROD Activity

**Figure 3:**
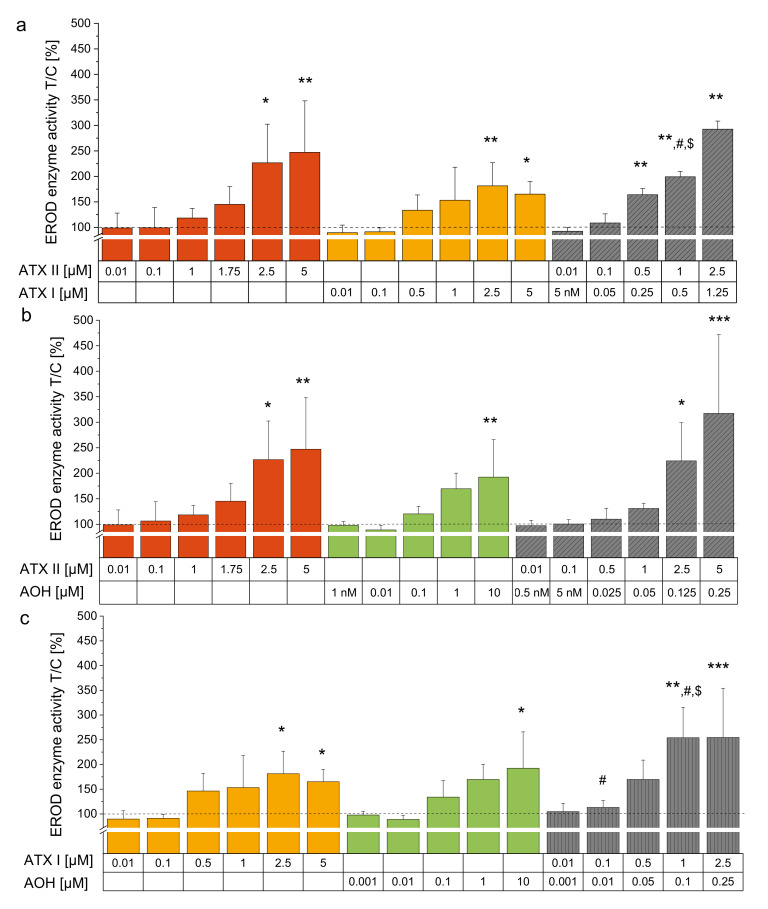

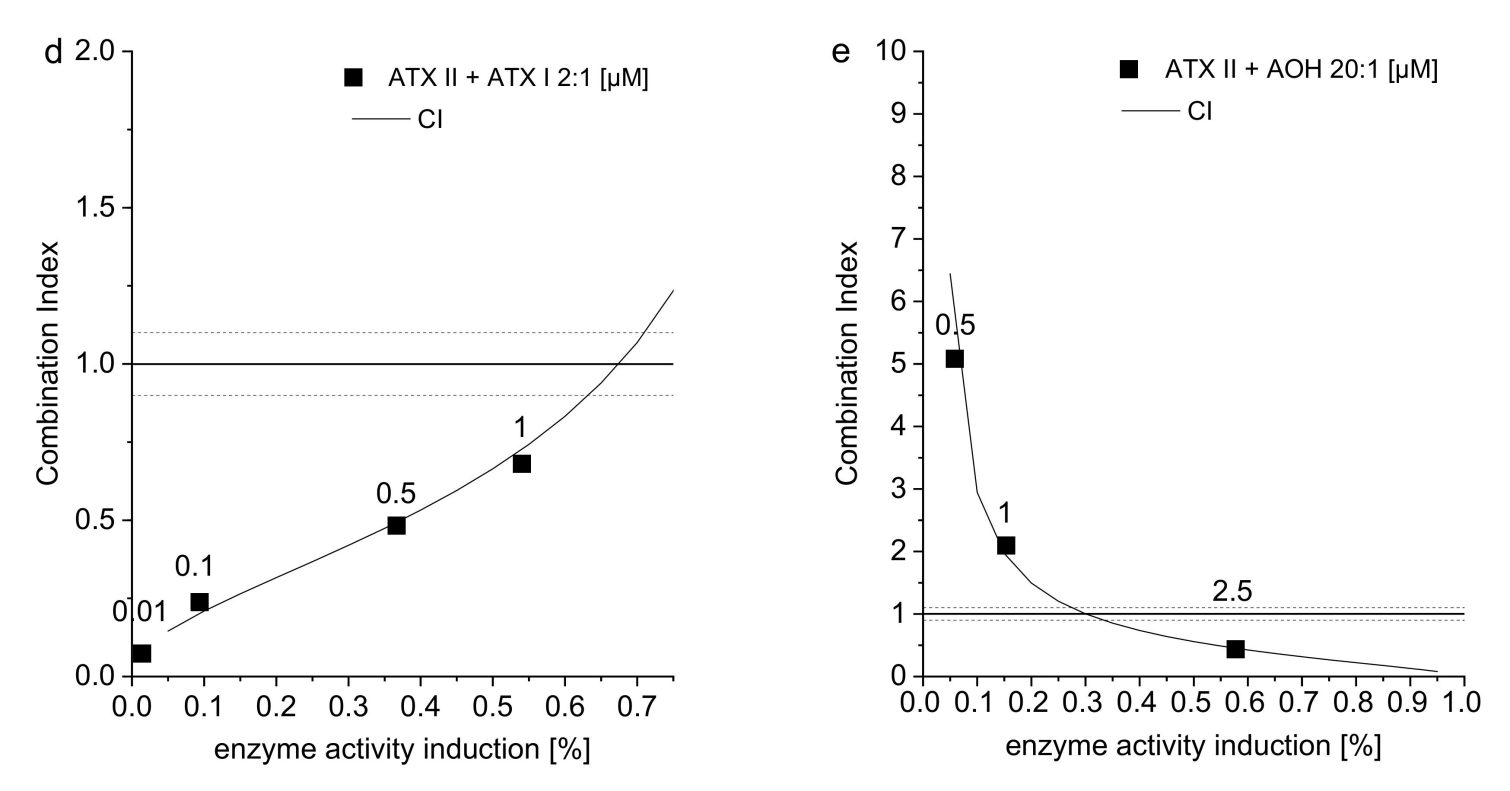
EROD assays with binary mixtures of AOH, ATX-I, and ATX-II. Bar diagrams show EROD enzyme activity measurements (pmol resorufin/mg protein*min) expressed as means + SD of at least 3 individual experiments in relation to the solvent control (1% DMSO). (**a**) ATX-II and ATX-I alone and in combination (ratio 2:1), (**b**) ATX-II and AOH (combinatory ratio 20:1) and (**c**) ATX-I and AOH (combinatory ratio 10:1). In order to approach the ratios resembling the extract, some concentrations used for the combinations diverge from the amounts applied singularly. “*” (*p* < 0.05), “**” (*p* < 0.01) and “***” (*p* < 0.001) indicate significant differences of incubation conditions against the NOEL. Significant differences were calculated applying a one-way ANOVA followed by Bonferroni post-hoc testing. “#” and “$” represent significant differences of combinations compared to the corresponding first and second single substance within the graph, respectively. (**d**,**e**) CI plots highlight combination index values at measured concentrations. Dotted lines indicate the “nearly additive effect corridor”.

**Figure 4:**
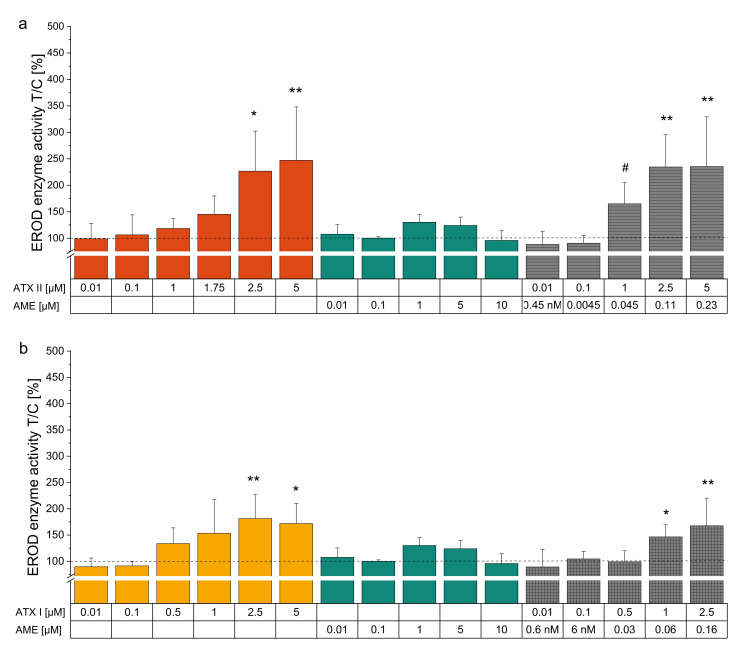
EROD assay of binary mixtures of ATX-II or ATX-I with AME in accordance to their respective ratios within the extract. Bar diagrams show EROD enzyme activity measurements (pmol resorufin/mg protein*min) presented as means + SD related to solvent control (1% DMSO) of at least 3 individual experiments. (**a**) ATX-II and AME binary combination at a ratio 22:1. (**b**) ATX-I and AME binary combination at a ratio 16:1. Significant differences between combinatory effects and ATX-I were calculated applying Student’s *t*-test and are indicated as “#” (*p* < 0.05). “*” highlights significant differences compared to the NOEL calculated via one-way ANOVA followed by Bonferroni post-hoc testing at *p* < 0.05 and “**” at *p* < 0.01. *n* = 3–5 individual experiments.

To shed more light on possible interactions of the single compounds regarding their impact on EROD enzyme activity, we tested binary mixtures. For this purpose, single substances were mixed together in ratios resembling their proportionality within the complex *Alternaria* extract (Table 1). Throughout the dose-range applied the binary combination of ATX-II und ATX-I in a 2:1 mixture revealed a synergistic interaction as a combinatory effect (Figure 3a,d). Combination index analysis exhibited a calculated CI_50_ of 0.66, indicating a synergistic interaction. Of note, mixture concentrations of ATX-I and ATX-II similar to the doses within 20 µg/mL of CE converged to the EROD activity enhancement of the extract (0.5 µM ATX-II + 0.25 µM ATX-I yielded 164 ± 12.2% enzyme activity, 20 µg/mL CE reached 193 ± 58.9% enzyme activity). For a 20:1 mixture of ATX-II and AOH, low doses showed an antagonistic interaction, whereas with higher concentrations this phenomenon switched towards a synergistic effect at the EC_50_ (Figure 3b,e). For this combination, a CI_50_ of 0.56 could be calculated. The combination of ATX-I and AOH at 10:1 (Figure 3c) exhibited an enhancement of EROD activity compared to ATX-I alone, with concentrations of 0.1 µM and 1 µM ATX-I, combined with AOH at 0.01 and 0.1 µM, respectively, showing the most prominent impact. The highest concentrations of ATX-I alone and in combination with AOH led to a plateau effect of the inducible EROD enzyme activity. Therefore, a calculation of the CI_50_ according to Chou and Talalay [52,59] was not feasible for this particular binary mixture. When combined with ATX-II or ATX-I, AME led to no distinct alterations of EROD enzyme activity (Figure 4).

### 3.3. Cell Metabolic Activity

**Figure 5:**
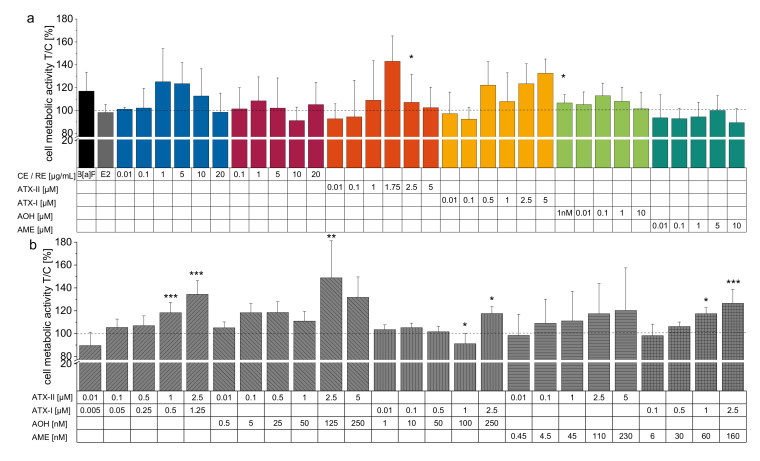
CellTiter-Blue^^®^^ assay. Metabolic activity measurements after 24 h of incubation presented as means + SD in comparison to solvent control (1 % DMSO) of at least 3 individual experiments. Metabolic activity of MCF-7: (**a**) 1 µM B[a]P, 1 nM E2, CE (blue) 0.01 µg/mL–20 µg/mL, RE (red) 0.1 µg/mL, single toxins ATX-II 0.01 µM–5 µM, ATX-I 0.01 µM–5 µM, AOH 1 nM–10 nM and AME 0.01 µM–10 µM and (**b**) binary mixtures of toxins (ATX-II + ATX I 1:0.5, ATX-II + AOH 1:0.05, ATX-I + AOH 10:1, ATX-II + AME 22:1 and ATX-I + AME 16:1) after 24 h of incubation. A negative control applying 0.1% TX-100 was conducted and successfully diminished cell metabolic activity (not shown in data). Significant differences were calculated utilizing one-way ANOVA against the NOEL followed by Bonferroni post-hoc testing and are represented by “*” (*p* < 0.05), “**” (*p* < 0.01) or “***” (*p* < 0.001).

Metabolic activity of treated cells was not compromised by the two versions of the *Alternaria* extract, any single compounds or mixtures of toxins at each of the concentrations used apart from ATX-I 1 µM mixed with AOH 100 nM. Instead, the perylene quinones ATX-I and ATX-II led to evident enhancements of metabolic activity at 5 µM and 1.75 µM, respectively. The combination of these two toxins dose-dependently increased metabolic activity. Besides, every other mixture except ATX-II combined with AME augmented metabolic activity in the high concentration ranges (Figure 5).

## 4. Discussion

The complex *Alternaria* toxin mixtures applied in this study were previously shown to exhibit anti-estrogenic properties in Ishikawa endometrial cancer cells [54]. This effect was linked to crosstalk of the ER and AhR.

Thus, the study at hand investigated the potential of complex *Alternaria* toxin mixtures as well as selected single toxins in corresponding binary mixtures to affect CYP 1 enzyme activity in estrogen-sensitive MCF-7 mammary cancer cells. The induction of both CYP 1A1 mRNA transcription and the according enzymatic activity was reported to serve as indicators for AhR activation [60]. Exhibiting a roughly 7-fold induction of EROD enzyme activity, 1 µM B[a]P served as a positive control for AhR signalling pathway activation (Figure 2a). In contrast, E2 showed no estrogen-related induction of CYP 1 activity in our MCF-7 cell model (Figure 2a), which was suggested in a previously published study [61]. Both, the CE and the RE (stripped from the two perylene quinones ATX-II and STTX-III), led to dose-dependent enhancements of CYP 1A1 activity (Figure 2a). This is in line with a previous report of the CE to increase CYP 1A1 expression levels in Ishikawa cells at concentrations comparable to the extracts EROD enzyme activity induction in MCF-7 [54]. Notably, the RE’s capability to induce CYP 1 activity surpassed the potent activity of the CE at higher concentrations. ATX-II and STTX-III are known for their mutagenicity and DNA strand breaking capacities and were shown to play major roles in the genotoxicity of complex *Alternaria* toxin mixtures [26,62,63]. Nonetheless, the distinctive EROD activity induction seen for the CE at concentrations encompassing concentrations of ATX-II previously reported exhibiting profound genotoxic capacities [26] was not evidently compromised compared to the RE’s potency. Besides, also the RE was previously found to exhibit DNA strand breaking and anti-estrogenic potential in Ishikawa cells, albeit to a lesser extent in comparison to the CE [54].

Single known extracts’ constituents, such as ATX-II and AOH were previously shown to activate the AhR, increase CYP 1A1 enzyme activity, which was shown via EROD assay and impact CYP 1A1 mRNA transcription at corresponding concentrations in KYSE 510 oesophageal cancer cells, while AME exhibited its potency primarily in HepG2 cells [51]. Testing single toxins revealed dose-dependent enzyme activation for ATX-I, ATX-II and AOH; in contrast, no evident induction of CYP 1 enzyme activity was exerted by AME (Figure 2b). Distinctive EROD enzyme activity induction was apparent at concentrations exceeding the corresponding presence of the single *Alternaria* toxins within the complex extracts at the amounts used for this study. Previously, ATX-II was found to increase CYP 1 transcription and activity in oesophageal cancer cells starting at a concentration of 0.05 µM and 0.1 µM, respectively [51], indicating a higher sensitivity to AhR activation compared to the breast cancer cell line MCF-7. ATX-I exerted a marginally, yet observably inferior potency towards EROD enzyme activity compared to ATX-II. Possibly, the highly reactive epoxide group, which ATX-I is lacking, could be held accountable for any discrepancies in effectiveness. This structural difference was previously shown to impact the genotoxic properties of the different perylene quinones in the cell lines Caco-2, HCT 116, HepG2, and V79 revealing ATX-II to exhibit 20-fold higher DNA-strand breaking potential compared to ATX-I [64]. Moreover, a previously uncovered divergence in the capability to mediate Nrf2 activation for ATX-II in contrast to ATX-I in colon carcinoma cells was justified by the sulfhydryl reactivity of the former’s epoxy moiety [65]. Besides, ATX-II was observed to be rather unstable under physiological conditions [66] and its chemical and metabolic stability was suggested to be decreased due to its epoxide group as opposed to ATX-I [67]. Nonetheless, the obtained results suggest a comprehensive capability to activate the AhR for the perylene quinone scaffold. Previously, 10 µM AOH were reported to induce EROD activity of KYSE 510 oesophageal cancer cells. This enhancement of CYP 1 enzyme activity was linked to AhR activation. In contrast, AOH was not capable of inducing EROD activity in HT29 colon carcinoma cells and HepG2 hepatoma cells [51]. Preceding published experiments showed inconclusive efficacy of AME towards CYP 1 enzyme activity alteration, increasing EROD activity in HepG2 cells at 10 µM and in KYSE 510 at 50 µM. This effect was diminished again at 50 µM in HepG2, possibly due to the onset of cytotoxicity. In addition, no increase in enzymatic activity was detected in HT29 cells [51]. This particular cell line lacks ERα [68], a receptor previously demonstrated as necessary for Ah-responsiveness in triple-negative breast cancer cells MDA-MB-231 [69]. Variations in EROD enzyme activity induction could also be owed to varying metabolism strategies exerted by the cell lines. For instance, DNA damage induced by one hour of incubation of AOH and AME was reduced after 3 h in HT-29 cells and associated with the cells’ capacities to exert phase II metabolism via glucuronidation of the two toxins [14,17,70]. Furthermore, cells of different origin bear variable expression levels of AhR, while additionally, various AhR ligands were reported to exert agonistic or antagonistic activities in a cell- and tissue-specific manner [71,72]. Besides, reduced bioactive effects of AME in comparison to AOH have been reported in the context of oxidative stress [17] and were attributed to the methyl group that distinguishes the two dibenzo-α-pyrones.

Combinatory effects of co-occurring substances may be exerted in several manners and are of toxicological relevance depending on their nature. Intriguingly, combining ATX-I and ATX-II exhibited an EROD enzyme activation potential beyond the single substances. Combinatory index analysis revealed a synergistic interaction between the two perylene quinones almost reaching the potency of the respective CE concentration (Figure 3a,d). Food surveys suggest a comparably low human exposure to perylene quinone derivates [73]. Nonetheless, exposure data on key toxins such as ATX-II is still scarce and needed for a proper risk assessment [7,74]. Moreover, ATX-I is described as a secondary metabolite of *Alternaria* spp. itself, but was further suggested to be a metabolite of ATX-II *in vivo*, *in vitro* and *in planta* [30,64,74]. Therefore, the role of ATX-II and ATX-I in the context of combinatory bioactive mechanisms within the plethora of *Alternaria* toxins should not be underestimated. The combination of ATX-II and AOH displayed an antagonistic interaction in the lower concentration range (corresponding to the applied concentrations of the CE), while it switched toward synergism in higher concentrations (Figure 3b,e). ATX-I combined with AOH evidently showed a combinatory effect, although combinatory index analysis could not be applied; therefore, a precise specification as additivity or synergism is not possible (Figure 3c). Lacking consistent impact on the perylene quinones’ EROD enzyme activating capacities (Figure 4a,b), a rather limited role in combinatory effects could be observed for AME.

Previously, AME and AOH were reported to exert xenoestrogenic properties *in silico* and *in vitro*, while the estrogenic potency of AME was considered more pronounced [75]. Unidirectional inhibitory AhR-ER crosstalk was demonstrated for TCDD in MCF-7 cells [76]. Upon AhR activation, proteasome-dependent degradation of AhR and ERα was induced without affecting new protein synthesis. Besides, a combination of AOH and AME was recently shown to also exhibit anti-estrogenic effects towards Ishikawa cells [54], albeit only at concentrations beyond the tested extracts’ ranges. Therefore, the anti-estrogenic capacities of the *Alternaria* extracts could not be satisfyingly pinpointed to the two dibenzo-α-pyrones, indicating other constituents to play a role. As single toxin effects are distinct and the combinatory impact of ATX-I and ATX-II almost reached the CE’s enzyme induction potency, our results suggest a key role for the two perylene quinones within the complex network of AhR- and ER-related signalling pathways. The above-described interdependencies of AhR and ER upon either activation might explain discrepancies in various cell lines; furthermore, the relevance for possible adverse effects in hormone-sensitive tissues is highlighted. Besides, extensive research revealed that AhR pathway activation disparately impacts mammary tumorigenesis and assigned a major role in breast cancer progression towards metastasis to AhR activation [77]. Of note, B[a]P was shown to elevate CYP 1A1 and CYP 1B1 mRNA in MCF-7 cells; therefore, enhanced EROD enzyme activity cannot exclusively be ascribed to a single CYP 1 enzyme as 7-ER poses a substrate for several above mentioned isoforms [78]. Nonetheless, previous studies on AOH, AME, and ATX-II as well as on the *Alternaria* extracts used showed CYP 1A1 mRNA enhancements in various cell lines [51,54]. Therefore, a high correlation of EROD enzyme activity towards CYP 1A1 could be assumed for the toxins and mixtures applied here as well. In light of previous studies suggesting single *Alternaria* toxins to be differently metabolized by varying cell lines, a contribution of the metabolism of the compounds by MCF-7, thus additionally enhancing EROD enzyme activity, cannot entirely be excluded for the interpretation of the combinatory data [51,70].

Metabolic activity measurements (measured as CellTiter^®^ Blue assay) applying the extracts or single constituents revealed no compromising potential. Occasional enhancements could be observed, especially for the two perylene quinones singularly and in binary mixtures (Figure 5). Previously, fluorescence resulting from the reduction of resazurin was reported to depend on cell growth [79] or mitochondrial, cytosolic, or microsomal enzymes [80]. Assays relying on resazurin reduction are often utilized to tie a connection between fluorescence and cell numbers [81]. Nonetheless, obtained protein content measurements applying the BCA assay after EROD activity assessment revealed no significant proliferation due to the compounds applied for most of the conditions (data not shown, Supplementary Figure S1). Apart from 5 µM ATX-I and the combinatory effect of ATX-I (1.25 µM) and ATX-II (2.5 µM) any increase in CellTiter-Blue^®^ fluorescence values could, therefore, be ascribed to enhanced metabolic activity due to the *Alternaria* toxins used in this study. Besides, a recent study revealed the ability of ATX-II to induce mitochondrial superoxide production in THP-1 derived macrophages [28], indicating enhanced mitochondrial activity, although this could not be demonstrated for ATX-I. A previous cytotoxicity study on combinations of ATX-II and AOH reported additivity towards mitochondrial activity, enhancing metabolic activity in HT29, HepG2, and HCEC-1CT cells applying the WST-1 assay. Antagonistic interactions could only be observed at concentrations in the two-digit micromolar range exhibiting decreased mitochondrial activity [82].

In summary, this study enlightens interactions of co-occurring *Alternaria* mycotoxins towards the AhR pathway in hormone-sensitive tissues involved in phase-I-metabolism of xenobiotics and endogenous substances. Nonetheless, further interference by other compounds not tested (or not identified) within the complex mixture affecting the CYP 1 enzyme activity needs to be considered for interpretation of the current data.

## 5. Conclusions

Taken together, the present study provides insights into the ability of naturally co-occurring *Alternaria* toxins like ATX-I, ATX-II, AOH, and AME to induce the activity of enzymes involved in phase I metabolism in complex and binary mixtures. We could decipher the two perylene quinone derivates ATX-I and ATX-II, as well as the dibenzo-α-pyrone AOH, as participants towards the CYP 1A1 activity induction exerted by complex *Alternaria* extracts in mammary cancer cells and their combinatory effects to be major contributors. Synergistic interactions between ATX-I and ATX-II, as well as interactions between AOH and the two altertoxins, respectively highlight the importance of consideration for combinatory effects in toxicological risk assessment for food safety. Within hormone-sensitive tissues, major enhancements of enzymes involved in phase I metabolism might be of toxicological concern considering their role in the metabolic activation of endogenous hormones and xenobiotics.

## Figures and Tables

**Table 1:** Single *Alternaria* toxin concentrations corresponding to applied concentrations of the CE.

	CE 0.01 µg/mL	CE 0.1 µg/mL	CE 1 µg/mL	CE 5 µg/mL	CE 10 µg/mL	CE 20 µg/mL
ATX-II [nM]	0.3	4	27	135	270	540
ATX-I [nM]	0.2	2	19	94	188	376
AOH [nM]	0.02	0.2	2	11	21	41
AME [nM]	0.02	0.2	1.5	8	16	32

## Supplementary Materials

The following are available online at https://www.mdpi.com/2218-273X/10/7/1018/s1, Figure S1: BCA assay after EROD measurements of 24 h of incubation.
