# Supplementary material for: Alternaria alternata Toxins Synergistically Activate the Aryl Hydrocarbon Receptor Pathway In Vitro
Source: Biomolecules. 2020 Jul 9;10(7):1018. doi: 10.3390/biom10071018 (PMC7407958; doi:10.3390/biom10071018)
Supplement: Supplementary file 1 [file biomolecules-10-01018-s001.pdf]

biomolecules

Supplementary Figures

Alternaria alternata Toxins Synergistically Activate the Aryl Hydrocarbon Receptor Pathway In Vitro

Julia Hohenbichler [1], Georg Aichinger[1], Michael Rychlik[2], Giorgia Del Favero[1], Doris Marko[1*]

[1] Department of Food Chemistry and Toxicology, University of Vienna; julia.hohenbichler@univie.ac.at; georg.aichinger@univie.ac.at; giorgia.del.favero@univie.ac.at;
[2] Chair of Analytical Chemistry, Technical University of Munich; michael.rychlik@tum.de;
* Correspondence: doris.marko@univie.ac.at

Received: 18 June 2020; Accepted: 30 June 2020; Published: date

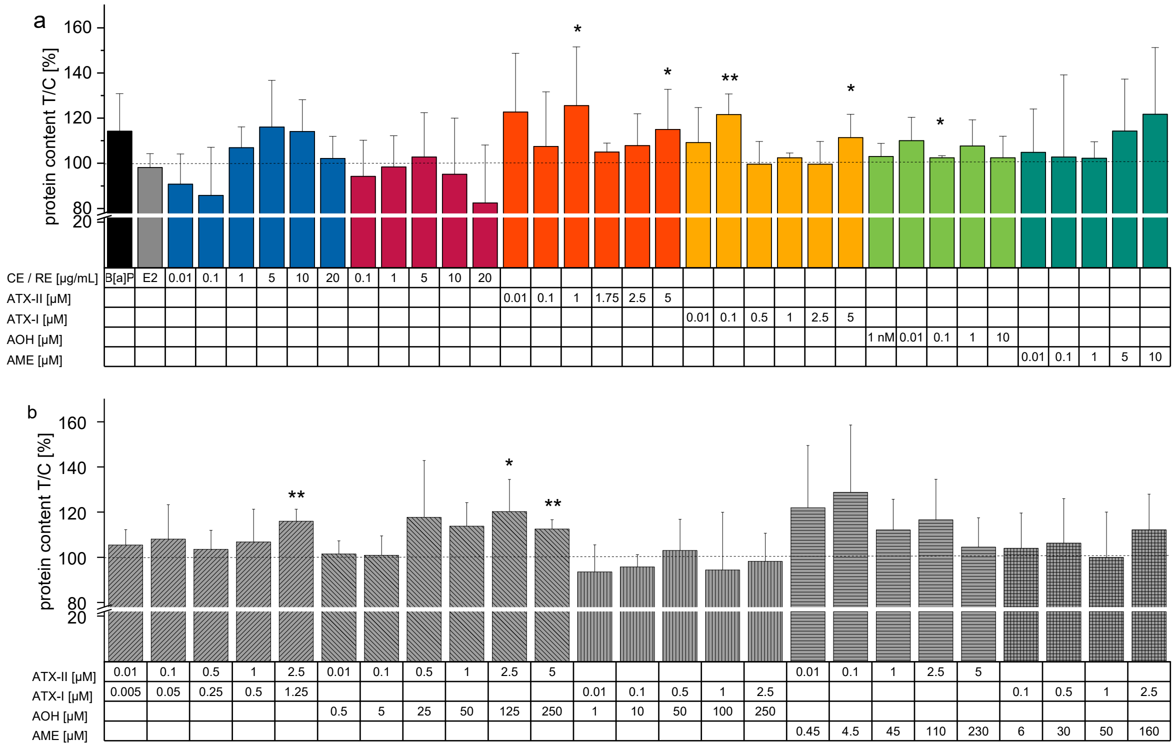

Supplementary Figure 1. BCA assay after EROD measurements of 24 hours of incubation. Protein content of each condition is represented as mean + SD compared to solvent control (1% DMSO). **(a)** protein content after incubations applying 1 µM B[a]P, 1 nM E2, CE (blue) and RE (red) and single toxins **(b)** protein content after combinatory incubations. Applying students' *t*-test, "*" (p<0.05), "**" ($p < 0.01$) and "***" ($p < 0.001$) indicate significant differences compared to solvent control.
